# Differential Gene Expression Profile of Human Neutrophils Cultured with *Plasmodium falciparum*-Parasitized Erythrocytes

**DOI:** 10.1155/2018/6709424

**Published:** 2018-07-05

**Authors:** Mohamad Alaa Terkawi, Ryo Takano, Kentaro Kato

**Affiliations:** ^1^National Research Center for Protozoan Diseases, Obihiro University of Agriculture and Veterinary Medicine, Inada-cho, Obihiro, Hokkaido 080-8555, Japan; ^2^Department of Orthopedic Surgery, Faculty of Medicine and Graduate School of Medicine, Hokkaido University, Kita-15, Nish-7, Kita-ku, Sapporo 060-8638, Japan; ^3^Research Center for Global Agromedicine, Obihiro University of Agriculture and Veterinary Medicine, Inada-cho, Obihiro, Hokkaido 080-8555, Japan

## Abstract

Neutrophils (PMNs) are the most abundant cellular component of our innate immune system, where they play central roles in the pathogenesis of and resistance to a broad range of diseases. However, their roles in malarial infection remain poorly understood. Therefore, we examined the transcriptional gene profile of human PMNs in response to *Plasmodium falciparum*-parasitized erythrocytes (iRBCs) by using oligonucleotide microarrays. Results revealed that PMNs induced a broad and vigorous set of changes in gene expression in response to malarial parasites, represented by 118 upregulated and 216 downregulated genes. The transcriptional response was characterized by the upregulation of numerous genes encoding multiple surface receptors, proteins involved in signal transduction pathways, and defense response proteins. This response included a number of genes which are known to be involved in the pathogenesis of malaria and other inflammatory diseases. Gene enrichment analysis suggested that the biological pathways involved in the PMN responses to the iRBCs included insulin receptor, Jak-STAT signaling pathway, mitogen-activated protein kinase (MAPK), and interleukin and interferon-gamma (IFN-*γ*) signaling pathways. The current study provides fundamental knowledge on the molecular responses of neutrophils to malarial parasites, which may aid in the discovery of novel therapeutic interventions.

## 1. Introduction

Malaria is one of the biggest public health problems globally, putting half of the world's population at risk of infection and killing nearly 0.5 million people annually. *Plasmodium falciparum* is the deadliest species of the parasite and is transmitted to humans by the mosquito *Anopheles gambiae* [1]. Once within the human body, the parasites initiate the clinically silent liver stage and the blood stage, which is associated with the clinical manifestations. In severe infections, *P. falciparum*-parasitized erythrocytes (iRBCs) sequestrate in the blood microvessels causing disruption of the microvascular blood flow to vital organs. Other accompanying events in malaria pathophysiology include leukocyte adhesion to the microvasculature, systemic production of inflammatory cytokines, and cytotoxic lymphocyte activation. Severe malaria is more common in children and nonimmune individuals who may develop anemia, jaundice, systemic inflammation, metabolic acidosis, renal failure, respiratory distress, and cerebral or placental disorders. Repeated exposure to the infections elicits robust immunity that usually protects from severe infection and death [1–3]. Therefore, detailed knowledge on the mechanisms by which immune cells regulate the host immunity to and immunopathogenesis of malaria may aid in the development of novel control strategies.

Neutrophils are polymorphonuclear leukocytes (PMNs) that are generated in the bone marrow from myeloid precursors and live for only a short time in the circulation. They represent the most abundant leukocyte population in the blood that rapidly infiltrates sites of inflammation or infection, where they eliminate the invading microorganisms [4]. PMNs eliminate pathogens intracellularly after phagocytizing them within their phagosomes using NADPH oxidase-dependent mechanisms and antibacterial proteins such as cathepsins, defensins, lactoferrin, and lysozymes. They can also eliminate microorganisms extracellularly by releasing neutrophil extracellular traps (NETs) that comprise a DNA element, antimicrobial histones, and proteases. In addition to their protective role, activated PMNs contribute to the pathogenesis of many inflammatory diseases by secreting an array of cytokines, chemokines, lipid mediators, myeloperoxidase, matrix metalloproteinases, and leukotrienes that mediate the initiation and progression of inflammation [4]. Yet, despite being indispensable for maintaining host health, the role of PMNs in host resistance to and pathogenesis of malarial infection remains controversial. For instance, numerous *in vitro* and *in vivo* studies have shown that neutrophils play a protective role against malarial parasites [5–10]. On the other hand, the contribution of neutrophils to the pathogenesis of cerebral malaria or to the severity of malarial infection has only been demonstrated in mice [11–13]. Moreover, a recent study has shown that depletion of PMNs and blocking of NETs prevent the development of malaria-associated acute lung injury/acute respiratory distress syndrome and significantly increase mouse survival [14]. Likewise, higher neutrophil numbers are present in peripheral blood of patients with severe malaria as compared to those in uncomplicated cases [15]. In the current study, therefore, we analyzed the transcriptional profiling of human PMNs in response to iRBCs by using oligonucleotide microarrays as a step toward understanding the role of cells in the immunopathogenesis of malaria. Results revealed that the transcriptional response of PMNs to iRBCs includes upregulation of numerous genes encoding multiple surface receptors and proteins involved in signal transduction pathways, defense, and inflammatory responses.

## 2. Materials and Methods

### 2.1. Ethics Statement

Our protocols for the use of human peripheral blood erythrocytes and PMNs were approved by the Research Ethics Review Committee of the Obihiro University of Agriculture and Veterinary Medicine (approval number 2014-01), and experiments were conducted in accordance with the Fundamental Guidelines for Academic Research Institutions under the jurisdiction of the Ministry of Education, Culture, Sports, Science, and Technology, Japan. Informed written consent to use the blood in this research was obtained from the donor.

### 2.2. Preparation of iRBCs and Neutrophils for Phagocytosis Assay

The *Plasmodium falciparum* 3D7 strain (Malaria Research and Reference Reagent Resource Center; MR4, American Type Culture Collection, Manassas, VA) was cultured and was enriched to ≥85% (Miltenyi Biotec, Auburn, CA, USA) for phagocytosis assay as previously described [16]. The PMNs were isolated from the peripheral blood of a healthy donor using Ficoll-Paque™ PLUS (GE Healthcare). After centrifugation, mononuclear layer cells, plasma, Ficoll, and interface layer of RBC pellets with Ficoll were discarded and RBC pellets were suspended in RBC lysis buffer (Sigma) to obtain PMNs. Purity of isolated cells was determined by performing a differential blood cell count of Giemsa-stained smears. Purified PMNs contained <1% other types of cells including lymphocytes, monocytes, eosinophils, and basophils. Freshly isolated PMNs (1 × 10^6^) were seeded onto poly-D-lysine-coated coverslips (Neuvitro, WA, USA) at 1 × 10^4^ and then cocultured at 37°C in a humidified atmosphere containing 5% CO_2_ with either uninfected RBCs or enriched iRBCs (1 : 20) for 1 h in the presence of 250 ng/ml human recombinant IFN-*γ* (R&D Systems). Cells were obtained at different time points from the same donor to minimize nonspecific reactions of neutrophils with the erythrocytes. All experiments on PMNs were carried out within 4 h after collection of blood. Control cells were cultured in the same medium supplemented with 250 ng/ml IFN-*γ* and uninfected RBCs (1 : 20). The percentage of phagocytosis was calculated based on the number of PMNs phagocytizing iRBCs or malaria pigment relative to the total number of PMNs on the coverslips stained with 10% Giemsa solution (Sigma) for 30 min [16]. To detect NETs, adherent PMNs were cocultured with iRBCs on glass coverslips for 1 h and then observed after immunostaining by means of confocal microscopy. Briefly, the coverslips were gently washed three times with PBS, fixed with 4% paraformaldehyde solution for 20 min at 4°C, and then treated with 0.3% Triton™ X-100 (Sigma) in PBS for 5 min for cell permeabilization. The primary antibody (anti-PfEXP2) for malarial parasite staining was diluted (1 : 1000) in PBS-3% fetal calf serum and incubated with the coverslips for 1 h at 37°C in a moist chamber. Alexa-Fluor® 488-conjugated goat anti-rabbit IgG (Molecular Probes, Invitrogen, Carlsbad, CA, USA) was used as the secondary antibody at a dilution of 1 : 400 to specifically label the first antibody for 30 min at 37°C. Hoechst was used to label the nuclear DNA and the NETs released from the cells (Molecular Probes). Finally, the coverslips were washed with PBS and mounted (Dako, Denmark) for analysis by a confocal laser scanning microscope (TCS NT, Leica, Heidelberg, Germany).

### 2.3. Microarray Analysis and Data Analysis

PMNs cultured with RBCs or iRBCs for 1 h were washed with ice-cold PBS and lysed with TRIzol Reagent (Invitrogen), and RNA samples were extracted using the RNeasy Plus Mini kit (Qiagen) according to the manufacturer's instructions. The experiment was performed in quadruplicate for each condition. High-quality RNA samples with integrity values of >7.0 (Agilent 2100 Bioanalyzer, Agilent Technologies) were subjected to microarray analysis [17]. Microarray data are available at the Gene Expression Omnibus (GEO) database (http:www.ncbi.nlm.nih.gov/geo/) under the accession number GSE114076.

### 2.4. Quantitative Real-Time Reverse Transcription Polymerase Chain Reaction (qRT-PCR)

cDNAs were synthesized from total RNA by using first-strand cDNA (Invitrogen) with oligo-dT primers, according to the manufacturer's instructions. The expression of target genes was analyzed by qRT-PCR as previously described [17]. The specific primers used in this study are listed in Supplementary Materials (Table S1).

### 2.5. Data Analysis

For the microarray data analysis, each gene expression array data set was normalized to the in silico pool for the neutrophils cocultured with RBCs. Differentially expressed genes with at least a 2.0-fold difference in expression that was statistically significant (*p* < 0.01) relative to the expression level in the neutrophils cocultured with RBCs were assigned to a Gene Ontology group to identify the biological functions that were enriched in the gene set. Statistically significant differences in gene expression between the PMNs cocultured with RBCs and those cocultured with iRBCs were determined by using a moderated *t*-test (*p* < 0.01). Enrichment of genes into GO terms for molecular function, biological processes, pathways, and interaction networks was achieved by using Genomatix GeneRanker (http://www.genomatix.de/), DAVID 6.7 (http://david.abcc.ncifcrf.gov), NET-GE (http://net-ge.biocomp.unibo.it/enrich), and Enricher (http://amp.pharm.mssm.edu/Enrichr/). Significant enrichment for each GO term was determined by a multiple testing correction method (*p* < 0.05).

## 3. Results and Discussion

**Figure 1:**
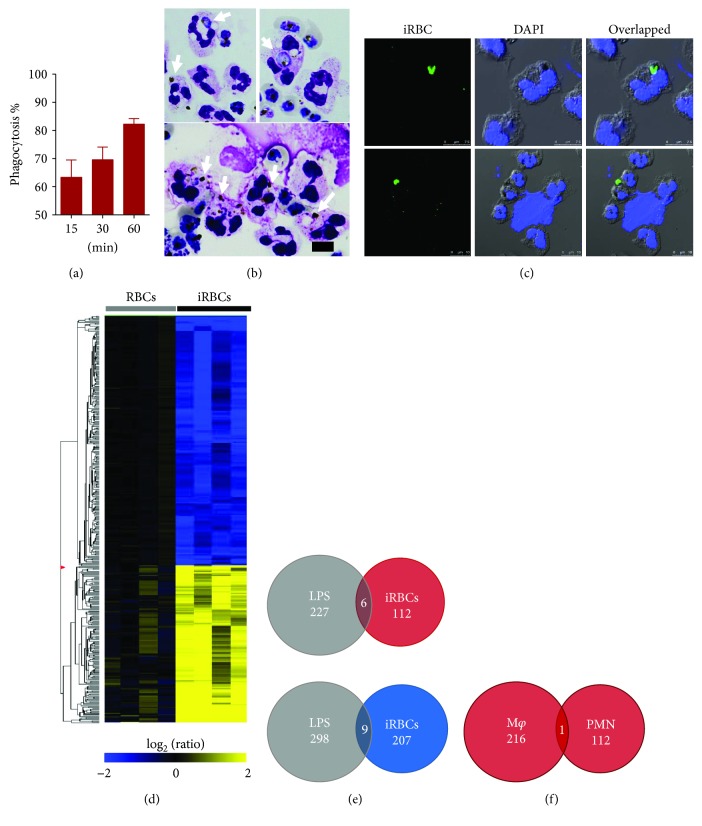
Response of human PMNs to iRBCs. (a) Percentage of PMNs phagocytizing iRBCs at different time points of incubation. Each bar represents the mean ± SEM for each group (*n* = 4). (b) Neutrophils phagocytizing iRBCs or malaria pigment as stained by Giemsa. Arrows indicate phagocytized iRBCs or pigment. Freshly isolated neutrophils were cultured with enriched iRBCs onto coverslips, and their phagocytic activity was examined at different time points after staining with Giemsa. (c) PMNs phagocytizing iRBCs (upper panels) and releasing NETs after coculture with iRBCs (lower panels). PMNs on the coverslips were stained with specific antibodies and examined by means of confocal microscopy. The scale bar is indicated on each image. (d) Gene profile of human neutrophils as assessed by microarray analysis. Clusters of downregulated genes and upregulated genes of neutrophils cultured for 1 h with human nonparasitized RBCs or enriched iRBCs (*n* = 4). (e) Venn diagram analyses for the comparison genes regulated in PMNs after stimulation with iRBCs or LPS. The upper panel is for upregulated genes, and the lower panel for downregulated genes. (f) Venn diagram analyses for the comparison genes regulated in macrophages and PMNs phagocytizing iRBCs.

Freshly isolated PMNs exhibited high phagocytic activity to iRBCs in medium supplemented with human noninfected serum and recombinant IFN-*γ*, which peaked within the 1st hour of culture (Figures 1(a) and 1(b)). Of note, a few NETs were observed in the PMN cultures with iRBCs but not in the PMN cultures with RBCs (Figure 1(c)). No phagocytic activity and NET formation were observed in neutrophil cultures with normal RBCs (data not shown). Surprisingly, phagocytic activity of these cells sharply declined in the 2nd hour of culture with iRBCs (data not shown). These results disagreed with earlier study reporting that activity of PMNs is dependent on the presence of malaria-immune serum in the medium [8]. The high phagocytic activity noted in our study can be explained by the fact that we used a high ratio of iRBCs in the assay, which increased the interaction with the cells and promoted phagocytic activity of PMNs. In addition, primed neutrophils by IFN-*γ* exhibit an elevation in their phagocytic activity *in vitro* in the absence of malaria-immune serum [6, 7]. Generally, the interaction between iRBCs with human peripheral blood mononuclear cells leads to a nonspecific stimulation of lymphocytes and a release of cytokines that facilitate and stimulate the interaction between iRBCs and other immune cells [18]. Therefore, addition of IFN-*γ* to PMN cultures is to mimic the priming status of PMNs during early-stage infection of malaria and to promote their interaction with iRBCs. Intraleukocytic iRBCs were observed after 15–30 min incubation, while intraleukocytic pigments were predominantly seen after 1 h incubation (Figures 1(a) and 1(b)). These results suggested that PMNs can rapidly lyse the phagocytized iRBCs within their phagolysosomes. This may explain the predominance of intraleukocytic pigments within PMNs in blood smears of field studies. Together, our findings are consistent with previous reports noting that the response of PMNs to iRBCs is rapid and includes phagocytosis and NET formation [7, 14, 19]. These data support the concept that PMNs rapidly respond to the invasion of protozoan parasites, including *Toxoplasma gondii* and *Leishmania amazonensis*, through promoting phagocytosis and NETosis [20–22].

To further gain insights into the molecular response of neutrophils phagocytizing iRBCs, we performed gene transcriptional analyses by using oligonucleotide-based DNA microarrays. Of 56,689 probes tested, 334 were statistically significantly differentially regulated (fold change > 2.0, *p* < 0.01); 118 and 216 probes were up- and downregulated, respectively, in PMNs cultured with iRBCs compared to the same probes in PMNs cultured with RBCs (Figure 1(d)). It is worth noting that a number of upregulated genes in PMNs phagocytizing iRBCs including BATF2, CISH, FCGR1A, SOCS3, CXCL10, HAS1, IRG1, IL1A, ELN, and CTSL are known to be involved in various inflammatory diseases [23]. To validate these transcriptional findings, the gene expression of randomly selected genes, including RIT2, MLLT10, CTSL, CYGB, ELN, SOCS3, IRG1, VIT, CISH, and CXCL10, was examined by qRT-PCR. The fold changes in gene expression determined by these two assays were somehow comparable with some differences in magnitude (Table 1).

To determine whether neutrophils in response to iRBCs elicit a specific gene expression profile, we compared our data to transcriptional microarray results of neutrophils stimulated by LPS [24]. Venn diagram analysis revealed that 6 upregulated genes (CISH, ERLIN1, FCRL1, IL1A, RPS6KA2, and SOCS3) and 9 downregulated genes (ADORA3, ARRDC3, BCOR, ERN1, FRAT1, HBB, KLHDC8B, TIGD3, and ZNF217) were present in the responses of neutrophils to either iRBC or LPS stimulation (Figures 1(d) and 1(e)). The discrepancies in the responses of PMNs to these stimuli are most likely due to distinct receptors and signaling pathways associated with each stimulus. Toll-like receptor 4 is the major LPS receptor that leads to the activation of the transcriptional regulator NF-*κ*B in neutrophils [25]. On the other hand, TLR7 seems to play a central role in early immune activation during malaria infection and is required for proinflammatory cytokine production and immune cell activation during the peak of parasitemia [26]. Given the possibility that the low percentage of the contaminating cells including monocytes in the PMN fraction may lead to incorrect results [27], we further compared the gene profile of PMNs to that of macrophages phagocytizing iRBCs [17]. Intriguingly, only the RIT2 gene was present in the responses of neutrophils and macrophages to iRBCs. However, the gene expression of RIT2 in PMNs was 156 times greater than that in macrophages phagocytizing iRBCs. Together, the transcriptional responses of PMNs associated with phagocytizing iRBCs included upregulation of surface receptors, signal transduction pathways, and inflammatory cytokines, which might reflect their roles in the pathogenesis during malaria infection.

**Figure 2:**
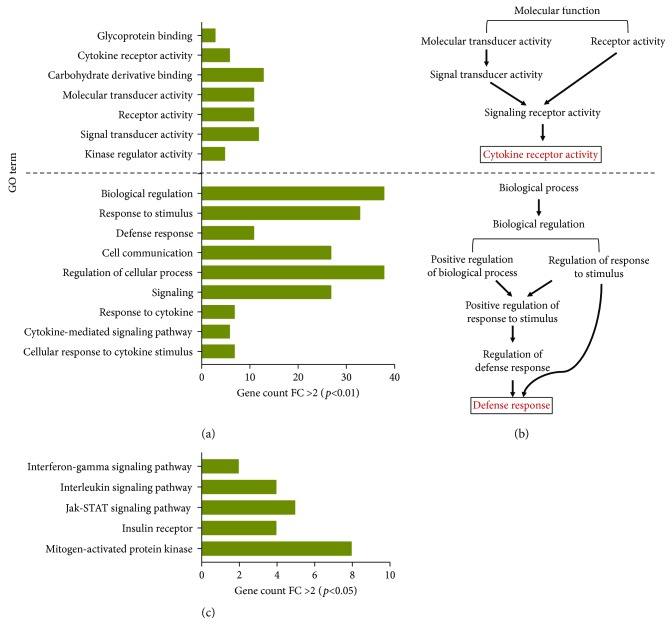
GO enrichment analyses of significantly differentially expressed genes of neutrophils in response to iRBCs. (a) Gene sets were categorized according to molecular function (upper panel) and biological processes (lower panel). (b) Network-based gene enrichment of the most significantly enriched terms for molecular function and biological processes. The annotation modules based on STRING for GO terms and multiple testing correction methods (*p* < 0.05) were performed based on NET-GE tools. (c) Biological pathways enriched in upregulated genes involved in the neutrophil response to iRBCs.

To further determine the functional and biological relevance of the regulated genes, enrichment analyses according to Gene Ontology (GO) specifications were carried out. Gene sets were categorized into multiple molecular functional groups, including kinase regulator activity, signal transducer activity, receptor activity, molecular transducer activity and carbohydrate derivative binding, cytokine receptor activity, and glycoprotein binding activity (Figure 2(a) and Table S2). Moreover, upregulated genes in PMNs phagocytizing iRBCs were categorized into multiple biological processes including cellular response to cytokine stimulus, cytokine-mediated signaling pathway, response to cytokine, signaling, regulation of the cellular process, cell communication, defense response, response to stimulus, and biological regulation (Figure 2(a) and Table S2). Of note, cytokine receptor activity and defense response were the most significantly enriched terms in PMNs phagocytizing iRBCs based on network enrichment analysis for molecular function or biological process, respectively (Figure 2(b)). Downregulated genes of PMNs phagocytizing iRBCs were mainly involved in oxygen binding, apoptosis, and metabolic processes (Table S3). Furthermore, functional pathway analysis was performed to define the relevant biological pathways involved in PMN responses to iRBCs. This analysis identified 5 statistically significant pathways including mitogen-activated protein kinase (MAPK), insulin receptor, Jak-STAT signaling pathway, interleukin signaling pathway, and interferon-gamma signaling pathway (Figure 2(c) and Table 2). In an analogous fashion, the IFN-*γ* signaling pathway was the most significantly enriched term in the gene signature of neutrophils infected with *Mycobacterium tuberculosis* [28], and MAPK, Jak-STAT, and interleukin signaling pathways are the most significantly enriched pathways in the primed human neutrophils by proinflammatory cytokines including TNF-*α* and GM-CSF [23]. On the other hand, the downregulated genes clustered in two pathways relevant to nuclear factor and lymphoid enhancer-binding factor (data not shown). To gain an insight into the regulation of signaling pathways involved in PMN response to iRBCs, transcription factor (TF) enrichment analysis for upregulated genes was performed using TF-protein-protein interactions (PPIs). Results showed that POU domain, class 4, transcription factor 1 (POU4F1: CCT3, MLLT10, MGA, KRT14, and FLNA) and POU5F1 (RIT2) are the most significantly (*p* = 0.01671 and 0.04158, resp.) enriched TF.

Our microarray data revealed that PMNs express a number of cell surface receptors for the recognition of iRBCs, including protein G receptors (P2RY4, GPR88, MRGPRG, FFAR3, HCAR1, and MRGPRF), adhesion receptors (VIT, CD69), sialic acid (SIGLEC12), Fc receptors (FCGR1a, FCRL1), FLNA, and IL-15RA. In general, priming of immune response to malaria is critically dependent on the activation of innate immune cells through their receptors including Toll-like receptors (TLRs), scavenger receptors, and the NOD-like receptor-containing pyrin domain 3 (NLRP3) inflammasome. Consequent activation of these receptors triggers distinct transcriptional pathways that lead to pathogen clearance [29, 30]. Our bioinformatics analysis revealed that the most upregulated genes of PMNs induced by iRBC phagocytosis included genes involved in MAPK signal transduction, which is known to be involved in cell proliferation, differentiation, stress responses, apoptosis, immune defense, and cytokine biosynthesis [29, 31]. Moreover, the gene profile of PMNs phagocytizing iRBCs indicated that the RIT2 gene was the top-regulated gene. RIT2, a member of the Ras superfamily, is a small molecular weight GTP-binding protein localized on the plasma membrane and is involved in the signaling of diverse biological processes including cell growth, differentiation, survival, senescence, and motility. The RIT2 gene is specifically expressed in neurons and plays a critical role in neuronal differentiation by mediating the magnitude and longevity of the MAPK cascade [32]. The regulation of RIT2 in neutrophils in response to iRBCs might probably sustain the activation of the MAPK signaling cascade needed for the senescence, motility, and cytokine production. Our next study is to elucidate the possible mechanism by which RIT2 maintains the phagocytic activity of neutrophils in response to malarial parasites. Our data corroborate previous findings from transcriptional analyses of whole-blood transcriptomes of Malawian patients infected with *P. falciparum* which revealed that the expression of numerous genes involved in MAPK signaling and inflammatory cytokine pathways is modified in response to *P. falciparum* exposure [15].

Other pathways that were upregulated in response of PMNs to iRBCs included interleukin and IFN-*γ* signaling pathways, which play an essential role in priming innate immune cells and promoting proinflammatory responses during malaria infection [33–35]. Nonetheless, excessive systemic production of inflammatory cytokines results in severe pathogenesis and poor outcome of infection [3, 35]. Our data showed the involvement of IL-1A, CXCL10, SOCS3, and CISH in the early response of PMNs to iRBCs, which are known to be associated with susceptibility to severe malaria infection [36–39]. Importantly, our previous study showed that macrophages phagocytizing iRBCs downregulate the expression of CXCL10 compared to control macrophages cultured with noninfected RBCs [34]. This may suggest that neutrophils but not macrophages are an important source of CXCL10, which is known to promote severe malaria by enhancing the recruitment of inflammatory cells and leukocyte activation [40]. On the other hand, SOCS3 and CISH are members of the suppressor of cytokine signaling (SOCS) family that mediate the regulation of cytokine receptor signaling and are implicated in a broad range of infectious diseases such as bacteremia and tuberculosis [41]. Together, our data showed that the gene profile of PMNs phagocytizing iRBCs is typified by the elevation of a number of transcripts involved in pathogenesis of malaria.

## 4. Conclusion

Our current study revealed that the responses of neutrophils to iRBCs are initiated by multiple surface receptors that consequently lead to the activation of distinct signaling pathways. This response includes the activation of a number of proinflammatory and regulatory cytokines, as well as of chemokines that may mediate the severity of the disease. These data improve our understanding of the early transcriptional events that occur in neutrophils in response to iRBCs and may aid in the identification of rational targets for future therapeutic interventions.

## Figures and Tables

**Table 1:** Changes in gene expression in neutrophils phagocytizing iRBCs as determined by DNA microarray analysis and qRT-PCR.

Gene	GenBank accession number	Fold change^†^	
		Microarray	qRT-PCR
RIT2	NM_002930	780.63	51.35 ± 14.44
MLLT10	NM_004641	287.92	6.20 ± 2.67
CTSL	NM_001912	87.55	41.10 ± 26.38
CYGB	NM_134268	39.87	9.65 ± 5.92
ELN	NM_000501	39.86	30.72 ± 19.00
SOCS3	NM_003955	37.19	6.90 ± 2.70
IRG1	NM_001258406	22.05	5.39 ± 1.80
VIT	NM_053276	18.94	8.41 ± 4.30
CISH	NM_145071	18.19	24.65 ± 3.22
CXCL10	NM_001565	17.69	10.83 ± 7.63
CD64	XM_005244958	10.56	8.24 ± 1.30

^†^Fold change indicates the mean expression level of the gene in neutrophils cocultured with *Plasmodium falciparum*-parasitized erythrocytes normalized to that in neutrophils cocultured with normal red blood cells.

**Table 2:** Enriched pathways for the upregulated genes in PMNs phagocytizing iRBCs.

Pathway	Count	*p* value	Genes
Mitogen-activated protein kinase (hsa04010)	8	0.000442	HCAR1, RPS6KA2, PEBP4, RIT2, MAPK8IP2, MLLT10, CDK5RAP1, HAS1
Insulin receptor (hsa04910)	4	0.000793	LETM1, CISH, RIT2, SOCS3
Jak-STAT signaling pathway (hsa04630)	5	0.000731	BATF2, CISH, SOCS3, MLLT10, CXCL10
Interleukin signaling pathway (P00970)	4	0.00139	IL1A, IL15RA, NOS3, RPS6KA2
Interferon-gamma signaling pathway (P00035)	2	0.02225	SOCS3, CISH

## Data Availability

The data used to support the findings of this study are available from the corresponding author upon request.

## Supplementary Materials

Supplementary Materials Table S1: primers used in this study. Table S2: GO terms for molecular function and biological process of the genes upregulated in PMNs phagocytizing iRBCs. Table S3: GO terms for molecular function and biological process of the genes downregulated in PMNs phagocytizing iRBCs.
