# Supplementary material for: Differential Gene Expression Profile of Human Neutrophils Cultured with Plasmodium falciparum-Parasitized Erythrocytes
Source: J Immunol Res. 2018 Jul 5;2018:6709424. doi: 10.1155/2018/6709424 (PMC6057315; doi:10.1155/2018/6709424)
Supplement: Supplementary Materials — Table S1: primers used in this study. Table S2: GO terms for molecular function and biological process of the genes upregulated in PMNs phagocytizing iRBCs. Table S3: GO terms for molecular function and biological process of the genes downregulated in PMNs phagocytizing iRBCs. [file 6709424.f1.pdf]

Differential gene expression profile of neutrophils phagocytizing *Plasmodium falciparum*-parasitized erythrocytes

Mohamad Alaa Terkawi[1,2], Ryo Takano[1], Kentaro Kato[1,3,]*

[1]National Research Center for Protozoan Diseases, Obihiro University of Agriculture and Veterinary Medicine, Inada-cho, Obihiro, Hokkaido 080-8555, Japan. [2]Department of Orthopedic Surgery, Faculty of Medicine and Graduate School of Medicine, Hokkaido University, Kita-15, Nish-7, Kita-ku, Sapporo, 060-8638, Japan.[3]Research Center for Global Agromedicine, Obihiro University of Agriculture and Veterinary Medicine, Inada-cho, Obihiro, Hokkaido 080-8555, Japan.

Supplementary Materials

Table S1. Primers used in this study.

Gene	Forward (5'→3')	Reverse (5'→3')
RIT2	AAGCACCTGACACGGGTG	GAGGGACCGGAGGAAAAAAA
MLLT10	CTACCCCTCTCACCCACACAA	TGGCATTGGATGTGTTGCA
CTSLR	ACAGGGAAGGGAAACACAGCT	TGTCTCCAAAGGCGTTCATG
CXCL10	CCAGAATCGAAGGCCATCAA	CCTTTCCTTGCTAACTGCTTTCA
CYGB	GGTCATTCTGGAGGTGGTCG	TCAGGTGGGAAGTCACTGGC
ELN1	GGTATCCCATCAAGGCCCC	AGTTTCCCTGTGGTGTAGGG
IRG1	TGAAGTGCAAGGCCGATTACT	GGCATGTCATTGGCCTCCT
SOCS3	ACCTTTCTGATCCGCGACAG	GCTGAGCGTGAAGAAGTGGC
CD64	AGAGGCCTGGTTTGCAGCTT	GGGTCTTGCTGCCCATGTAG
CISH	GAGGATCTGCTGTGCATAGCC	GCCAGATTCCCGAAGGTAGG
VIT	GGATGCCAAGACCCCAAATA	GGATGCATACACGTCAGTGCC
B-actin	CCTCACCCTGAAGTACCCCA	TCGTCCCAGTTGGTGACGAT

Table S 2. GO terms for molecular function and biological process of the genes upregulated in PMNs phagocytizing iRBCs.

GO terms	ID	Count	P value	Genes
**Molecular function**				
**Kinase regulator activity**	GO:0019207	5	0.0024	CXCL10, CDK5RAP1, CISH2, MAPK8IP2, SOCS3
**Signal transducer activity**	GO:0004871	12	0.012	CD69, FCGR1A, GPR88, MRGPRF, MRGPRG, FLNA, FFAR3, GABRQ, HCAR1, IL15R, KRT17, P2RY4, FFAR3
**Receptor activity**	GO:0004872	11	0.024	CD69, FCGR1A, GPR88, MRGPRF, MRGPRG, FLNA, FFAR3, GABRQ, HCAR1, IL15R, KRT17, P2RY4, TFR2
**Molecular transducer activity**	GO:0060089	11	0.024	CD69, FCGR1A, GPR88, MRGPRF, MRGPRG, FLNA, FFAR3, GABRQ, HCAR1, IL15R, KRT17, P2RY4, TFR2
**Carbohydrate derivative binding**	GO:0097367	13	0.028	CXCL10, RIT2, WNK4, CTSL, CCT3, FLNA, NMNAT1, NOS3, P2RY4, RPS6KA2, SEPHS2, TFR2, VIT
**Cytokine receptor activity**	GO:0004896	6	0.0345	CXCL10, SOCS3, CISH, IL15RA, IL1A, NOS3
**Glycoprotein binding**	GO:0001948	3	0.038	CTSL, FLNA, TFR2

**Biological process**				
**Cellular response to cytokine stimulus**	GO:0071345	7	0.00177	IL15RA, IL1A, CISH, FCGR1A, SOCS3, CXCL10, IRG1
**Cytokine-mediated signaling pathway**	GO:0019221	6	0.0023	IL15RA, IL1A, CISH, FCGR1A, SOCS3, CXCL10
**Response to cytokine**	GO:0034097	7	0.0041	IL15RA, IL1A, CISH, FCGR1A, SOCS3, CXCL10, IRG1
**Signaling**	GO:0023052	27	0.0044	P2RY4, IL15RA, HCAR1, FFAR3, CPNE6, IL1A, GABRQ, CNIH2, WNK4, CISH, RPS6KA2, FCGR1A, TFR2, SLC1A7, KRT17, RIT2, ERLIN1, GPR88, FLNA, MAPK8IP2, SOCS3, CTSL, CXCL10, MRGPRF, MRGPRG, ANKS1A, IRG1
**Regulation of cellular process**	GO:0050794	38	0.0067	P2RY4, IL15RA, HCAR1, FFAR3, CPNE6, IL1A, GABRQ, MGA, BATF2, SPIRE1, CNIH2, WNK4, CISH, RPS6KA2, HLX, FCGR1A, TFR2, FAM132B, KRT17, RIT2, ERLIN1, GPR88, FLNA, ZSCAN2, VIT, MAPK8IP2, SOCS3, MLLT10, CTSL, CDK5RAP1, CXCL10, IKZF4, HAS1, MRGPRF, CYGB, MRGPRG, ANKS1A, IRG1

**Cell communication**	GO:0007154	27	0.0075	P2RY4, IL15RA, HCAR1, FFAR3, CPNE6, IL1A, GABRQ, CNIH2, WNK4, CISH, RPS6KA2, FCGR1A, TFR2, SLC1A7, KRT17, RIT2, ERLIN1, GPR88, FLNA, MAPK8IP2, SOCS3, CTSL, CXCL10, MRGPRF, MRGPRG, ANKS1A, IRG1
**Defense response**	GO:0006952	11	0.0096	HCAR1, FFAR3, IL1A, BATF2, RPS6KA2, FCGR1A, MAPK8IP2, SOCS3, CTSL, CXCL10, IRG1
**Response to stimulus**	GO:0050896	33	0.0130	CXCL10, CDK5RAP1, ERLIN1, FCGR1A, GPR88, HLX, IKZF4, MRGPRF, MRGPRG, MGA, RIT2, WNK4, ANKS1A, BATF2, CTSL, CPNE6, CNIH2, CYGB, CISH, ELN, FLNA, FFAR3, GABRQ, HAS1, HCAR1, IL1A, IL15RA, KRT17, LETM1, MAPK8IP2, MLLT10, P2RY4, RPS6KA2, SPIRE1, SOCS3, TFR2, VIT, ZSCAN2
**Biological regulation**	GO:0065007	38	0.0170	CXCL10, ERLIN1, FCGR1A, GPR88, HLX, MRGPRF, MRGPRG, RIT2, WNK4, ANKS1A, BATF2, CTSL, CPNE6, CNIH2, CYGB, CISH, FLNA, FFAR3, GABRQ, HAS1, HCAR1, IL1A, IL15RA, KRT14, KRT17, MAPK8IP2, NMNAT1, P2RY4, RPS6KA2, SOCS3, TFR2

Table S 3. GO terms for molecular function and biological process of the genes downregulated in PMNs phagocytizing iRBCs.

GO terms	ID	Count	P value	Genes
**Molecular function**				
**Oxygen binding**	GO:0019825	5	2.71561E-05	HBA2, HBM, HBG1, HBD, HBB
**Organic cyclic compound binding**	GO:0097159	68	7.54699E-05	ZNF12, BCOR, MBD4, NEK6, KIAA0907, ZNF3, ZNF766, SP3, SMG6, DIDO1, STK17B, ZNF627, DOT1L, HBA2, ERN1, ZNF217, HBM, SNAI3, HBG1, ZNF592, CLK4, SYNJ2, RNASET2, ZNF518B, ZNF350, GPANK1, P2RY2, VSIG8, SF1, ALAS2, RHOB, STK17A, THUMPD3, ZNF322, ZNF100, RHOBTB2, DNAJA2, ATP7A, RAC1, OTX1, PLAGL2, KCTD12, LTB4R, PHC2, THAP1, HBD, CEBPE, POLM, TCEANC, BUD13, NLRC4, VEZF1, AKNA, DCAF13, HEY1, TIGD3, NKX3-1, NLRP12, SRXN1, HBB, RAB39B, AK3, ZFP36L2, SNAI1, NUAK2, HIC1, ZNF277, UBE2C

**Cysteine-type endopeptidase activator activity involved in apoptotic process**	GO:0008656	3	0.000361589	NKX3-1, NLRP12, CARD8
**Endonuclease activity**	GO:0004519	6	0.000364607	MBD4, SMG6, RPP38, ERN1, RNASET2, PELO
**Peptidase activator activity involved in apoptotic process**	GO:0016505	3	0.000499351	NKX3-1, NLRP12, CARD8
**Endoribonuclease activity**	GO:0004521	4	0.000649157	SMG6, RPP38, ERN1, RNASET2
**Cation binding**	GO:0043169	48	0.001988043	ZNF12, NEK6, ZNF3, ZNF766, SP3, SMG6, RNF144B, DIDO1, ZNF627, HBA2, ERN1, ZNF217, HBM, SNAI3, HBG1, ZNF592, ZNF518B, ZNF350, SF1, ALAS2, ZNF322, RRM2B, ZNF100, ZNF830, DNAJA2, ATP7A, PLAGL2, PHF12, TKTL1, PHC2, THAP1, HBD, CAPN15, POLM, NLRC4, VEZF1, THBD, CBLL1,

				INPP1, HBB, PELO, EFCAB1, ZFP36L2, SNAI1, NUAK2, HIC1, NOTCH2NL, ZNF277
**DNA binding**	GO:0003677	32	0.00217988	ZNF12, BCOR, MBD4, ZNF3, ZNF766, SP3, SMG6, ZNF627, DOT1L, ZNF217, SNAI3, ZNF592, ZNF518B, ZNF350, ZNF322, ZNF100, OTX1, PLAGL2, PHC2, THAP1, CEBPE, POLM, TCEANC, VEZF1, AKNA, HEY1, TIGD3, NKX3-1, ZFP36L2, SNAI1, HIC1, ZNF277
**Metal ion binding**	GO:0046872	47	0.002187932	ZNF12, NEK6, ZNF3, ZNF766, SP3, SMG6, RNF144B, DIDO1, ZNF627, HBA2, ERN1, ZNF217, HBM, SNAI3, HBG1, ZNF592, ZNF518B, ZNF350, SF1, ZNF322, RRM2B, ZNF100, ZNF830, DNAJA2, ATP7A, PLAGL2, PHF12, TKTL1, PHC2, THAP1, HBD, CAPN15, POLM, NLRC4, VEZF1, THBD, CBLL1, INPP1, HBB, PELO, EFCAB1, ZFP36L2, SNAI1, NUAK2, HIC1, NOTCH2NL, ZNF277
**Biological process**				
**Oxygen transport**	GO:0015671	5	1.10817E-07	HBA2, HBM, HBG1, HBD, HBB

**Cellular macromolecule metabolic process**	GO:0044260	92	3.25673E-07	NAT9, ZNF12, PILRB, DNAJC3, BCOR, FHOD1, MBD4, NEK6, ASB2, RGS3, ZNF3, ZNF766, SP3, SMG6, RPP38, RNF144B, ARRDC3, DIDO1, STK17B, ZNF627, DOT1L, PDRG1, ERN1, ZNF217, CLN3, SNAI3, ZNF592, SLC9A1, CLK4, SYNJ2, RNASET2, ZNF518B, ZNF350, SF1, ALAS2, ARRDC4, STK17A, ZNF322, RRM2B, NAB2, ZNF100, ZNF830, DNAJB5, DNAJA2, MGAT3, ATP7A, RAC1, OTX1, HECW2, PLAGL2, PHF12, DUSP28, PHLDA1, PHC2, AKTIP, TXNIP, THAP1, CAPN15, CEBPE, POLM, TCEANC, ENC1, BUD13, RFT1, NLRC4, VEZF1, TBCC, AKNA, DCAF13, BCDIN3D, HEY1, NKX3-1, CBLL1, ID2, DBNL, NLRP12, INPP1, PHLDA2, CARD8, PELO, ZFP36L2, TLE4, RPRD1A, SNAI1, NUAK2, HIC1, FADD, FUT4, STX1A, ZNF277, UBE2C, SERPINB8
**Cell death**	GO:0008219	32	2.64595E-05	DNAJC3, NEK6, RNF144B, DIDO1, STK17B, HBA2, ERN1, CLN3, SLC9A1, TNFRSF12A, RHOB, STK17A, RRM2B, ATP7A, RAC1, PLAGL2, PHLDA1, AKTIP, TXNIP, NLRC4, PNMA5, FAM134B,

				NKX3-1, DBNL, NLRP12, PHLDA2, HBB, CARD8, SNAI1, NUAK2, HIC1, FADD
**Apoptotic process**	GO:0006915	30	5.16586E-05	DNAJC3, NEK6, RNF144B, DIDO1, STK17B, ERN1, CLN3, SLC9A1, TNFRSF12A, RHOB, STK17A, RRM2B, ATP7A, RAC1, PLAGL2, PHLDA1, AKTIP, TXNIP, NLRC4, PNMA5, FAM134B, NKX3-1, DBNL, NLRP12, PHLDA2, CARD8, SNAI1, NUAK2, HIC1, FADD
**Cellular metabolic process**	GO:0044237	99	5.93162E-05	NAT9, ZNF12, PILRB, DNAJC3, BCOR, FHOD1, MBD4, NEK6, ASB2, RGS3, ZNF3, ZNF766, SP3, SMG6, RPP38, RNF144B, ARRDC3, DIDO1, STK17B, ZNF627, DOT1L, MID1IP1, HBA2, PDRG1, ERN1, ZNF217, CLN3, SNAI3, ZNF592, SLC9A1, CLK4, SYNJ2, RNASET2, ZNF518B, ZNF350, SF1, ALAS2, ARRDC4, STK17A, ZNF322, RRM2B, NAB2, ZNF100, ZNF830, DNAJB5, DNAJA2, MGAT3, ATP7A, RAC1, OTX1, HECW2, PLAGL2, PHF12, DUSP28, PHLDA1, TKTL1, PHC2, AKTIP, TXNIP, THAP1, CAPN15, CEBPE, POLM, TCEANC, ENC1, BUD13,

	RFT1, NLRC4, VEZF1, TBCC, AKNA, DCAF13, BCDIN3D, HEY1, FAM134B, NKX3-1, CBLL1, ID2, DBNL, NLRP12, INPP1, PHLDA2, HBB, CARD8, PELO, AK3, ZFP36L2, TLE4, COX19, RPRD1A, SNAI1, NUAK2, HIC1, FADD, FUT4, STX1A, ZNF277, UBE2C, SERPINB8
